# PM_2.5_-Related Health Economic Benefits Evaluation Based on Air Improvement Action Plan in Wuhan City, Middle China

**DOI:** 10.3390/ijerph17020620

**Published:** 2020-01-18

**Authors:** Zhiguang Qu, Xiaoying Wang, Fei Li, Yanan Li, Xiyao Chen, Min Chen

**Affiliations:** 1 Research Center for Environment and Health, Zhongnan University of Economics and Law, Wuhan 430073, China; quzhiguang@zuel.edu.cn (Z.Q.); wxy@stu.zuel.edu.cn (X.W.); ynli@stu.zuel.edu.cn (Y.L.); chenxiyao@stu.zuel.edu.cn (X.C.); 2 School of Information and Safety Engineering, Zhongnan University of Economics and Law, Wuhan 430073, China; 3 Key Laboratory of Virtual Geographic Environment (Ministry of Education), Nanjing Normal University, Nanjing 210023, China

**Keywords:** air quality, PM_2.5_, health economic benefits, BenMAP, Wuhan City

## Abstract

On the basis of PM_2.5_ data of the national air quality monitoring sites, local population data, and baseline all-cause mortality rate, PM_2.5_-related health economic benefits of the Air Improvement Action Plan implemented in Wuhan in 2013–2017 were investigated using health-impact and valuation functions. Annual avoided premature deaths driven by the average concentration of PM_2.5_ decrease were evaluated, and the economic benefits were computed by using the value of statistical life (VSL) method. Results showed that the number of avoided premature deaths in Wuhan are 21,384 (95% confidence interval (CI): 15,004 to 27,255) during 2013–2017, due to the implementation of the Air Improvement Action Plan. According to the VSL method, the obtained economic benefits of Huangpi, Wuchang, Hongshan, Xinzhou, Jiang’an, Hanyang, Jiangxia, Qiaokou, Jianghan, Qingshan, Caidian, Dongxihu, and Hannan District were 8.55, 8.19, 8.04, 7.39, 5.78, 4.84, 4.37, 4.04, 3.90, 3.30, 2.87, 2.42, and 0.66 billion RMB (1 RMB = 0.1417 USD On 14 October 2019), respectively. These economic benefits added up to 64.35 billion RMB (95% CI: 45.15 to 82.02 billion RMB), accounting for 4.80% (95% CI: 3.37% to 6.12%) of the total GDP of Wuhan in 2017. Therefore, in the process of formulating a regional air quality improvement scheme, apart from establishing hierarchical emission-reduction standards and policies, policy makers should give integrated consideration to the relationship between regional economic development, environmental protection and residents’ health benefits. Furthermore, for improving air quality, air quality compensation mechanisms can be established on the basis of the status quo and trends of air quality, population distribution, and economic development factors.

## 1. Introduction

In recent years, with the rapid development of industrialization and urbanization, the consumption of energy resources has continued to increase, and China is now suffering from a relatively severe situation of air pollution [1,2]. Facing the increasing pressure for air pollution prevention, the Chinese government promulgated and implemented the most stringent atmospheric pollution action plan in China’s history: the Action Plan of Air Pollution Control (hereinafter referred to as “Ten Measures for Air”) on 12 September 2013 [3]. Then, the main cities such as Beijing, Shanghai, Wuhan, and Chengdu enacted the Action Plan to improve air quality. For the 74 key cities of China, annual average concentration of PM_2.5_ (particulate matter with an aerodynamic diameter ≤2.5 μm) dropped by 34.7%, from 72 μg/m³ in 2013 to 47 μg/m³ in 2017 [4]. Despite the overall improvement in air quality throughout the country, there are still 64.2% of cities in which the annual average concentration of PM_2.5_ exceeds China’s second-level environmental air quality standards (35 μg/m³). The current situation of PM_2.5_ pollution in China is therefore still concerning [4,5,6].

Epidemiological studies show that PM_2.5_ has adverse effects on human health. For every 10 μg/m^3^ increase in PM_2.5_ concentration, the risk of all-cause, cardiopulmonary, and lung cancer mortality increased by approximately 4%, 6%, and 8%, respectively [7]. Even in areas with light air pollution and a more developed economy, the exposure to PM_2.5_ could increase the risk of resident mortality, and the strongest extra risk for death is increased by 0.74% (95% confidence interval (CI): 0.11–1.38%) for every 10 μg/m^3^ increase in PM_2.5_ concentration [8]. According to the 2015 Global Burden of Disease (GBD 2015) report, PM_2.5_ has become the fifth global death risk factor in the world, and the consequences of exposure to PM_2.5_ contributed to an estimated 4.2 million (95% uncertainty interval: 3.7–4.8 million) deaths globally [9]. Recently, in order to quantitatively evaluate the effects of air pollution prevention policy implementation, the health benefits of air quality improvement and their monetization values were studied in different countries on the basis of epidemiological studies [10,11,12,13,14,15]. Abel et al. quantified the health benefits of a 12% summertime (June, July, and August) reduction in baseload electricity demand in the United States on the basis of epidemiological studies and the corresponding reduction rate of PM_2.5_ (0.55%). Their results showed that the reduced exposure to PM_2.5_ annually avoided 300 premature deaths (95% CI: 60 to 580), valued at about USD 2.8 billion (USD 0.13 billion to USD 9.3 billion) [15]. In China, the implementation of Ten Measures for Air prevented about 60,213 residents from premature death, and the estimated increase in health benefits was approximately 54.97 billion Chinese yuan (RMB, 1 RMB = 0.1417 USD on 14 October 2019) [16]. Furthermore, Dai et al. quantitatively evaluated the public health benefits of Shanghai’s Clean Air Action Plan (2013–2017), and the results showed that the total health benefits realized by Shanghai were 11.841 billion RMB (95% CI: 50.24–178.19 billion RMB), accounting for 0.55% of Shanghai’s Gross Domestic Product (GDP) in 2013 (95% CI: 0.23%–0.82%) [17]. These studies suggested that global preventative PM_2.5_ pollution policies had public health effects and corresponding economic benefits to different extents.

In February 2014, as one of the first-phase monitoring and implementation cities of the new standard (No. GB3095–2012), the only mega city in the six central provinces of China, and the capital of Hubei province, Wuhan issued the Air Improvement Action Plan (Plan; 2013–2017). The Plan deployed appropriate measures such as strengthening pollution prevention, optimizing industrial structures, innovating environmental management mechanisms, and strengthening information disclosure to improve air quality (details are shown in Table S1) [18]. Since the implementation of the Plan, the annual average concentration of PM_2.5_ was decreased from 94 μg/m^3^ in 2013 to 53 μg/m³ in 2017 (by 43.6% decrease). The number of days with excellent (0 < Air Quality Index (AQI) ≤ 50) or good ambient air quality (51 < AQI ≤ 100) in Wuhan from 2013 to 2017 were 160, 182, 192, 237, and 255 d, respectively, showing a growing annually tendency (Details about AQI are shown in Tables S2 and S3) [19,20]. To our best knowledge, no scholars have studied the health economic benefits of improving the air quality in Wuhan from 2013 to 2017. In order to provide a quantitative cost–benefit analysis of the Plan and synthetically improve corresponding local environmental policies, it was important to investigate the health economic benefits of reducing PM_2.5_ concentration in Wuhan.

The major aims of this study were: (1) to estimate the annual average concentration of PM_2.5_ decrease in 13 districts in Wuhan from 2013 to 2017 by using monitoring data derived from nationally controlled sites and the Voronoi neighbor averaging (VNA) interpolation method; (2) to explore the avoided premature mortalities attributed to PM_2.5_ reductions for all districts in Wuhan on the basis of local demographic data, baseline all-cause mortality rate, and health impact functions; (3) to calculate the economic benefits of reducing PM_2.5_ concentration using the value of statistical life (VSL) method; (4) to evaluate the effectiveness and pitfalls of the Air Improvement Action Plan in Wuhan.

## 2. Materials and Methods

### 2.1. Study Area

The City of Wuhan, the provincial capital of Hubei Province, is an important industrial, science, and education base in China, a comprehensive transportation hub, a central Chinese tourism center city, and the core city of the Yangtze River Economic Belt. The city has a northern semitropical monsoon climate, and has four distinct seasons. The city has jurisdiction over 13 municipal districts with a total area of 8659.15 km^2^. Of these 13 municipal districts, Jiang’an District, Jianghan District, Qiaokou District, Hanyang District, Wuchang District, Qingshan District, and Hongshan District are central urban districts; while Dongxihu District, Hannan District, Caidian District, Jiangxia District, Huangpi District, and Xinzhou District are suburban districts. In recent years, the city has witnessed rapid urban expansion, and both the areas of built-up land and vegetation has increased [21]. In 2017, it had a population of over 10.89 million, regional GDP was 1341.03 billion RMB (ranking first in Central China), and the gross industrial output value above the designated size was 1443.33 billion RMB [22]. Moreover, the composition of primary, secondary, and tertiary industry was 3.0:43.7:53.3, and the per capita disposable income of residents was 38,642 RMB [22]. According to the official research in 2016 on the source identification of atmospheric particulate matter in Wuhan (http://www.hubei.gov.cn/gzhd/gzhd/hygq_49009/), results showed that the pollution contribution rates of industrial production, motor vehicle exhaust, coal burning, dust, and others (including biomass burning, living sources, agricultural sources) were 32%, 27%, 20%, 9%, and 12%, respectively. According to the statistics of the Wuhan Eco-Environment Bureau, in 2017, motor vehicles in Wuhan numbered 2.88 million, an increase of 11.27% compared to 2016 [23]. In recent years, days during which the city’s primary pollutant was PM_2.5_ accounted for more than 60% of the annual pollution days [19]. Facing the local pollution status of PM_2.5_ and its potential health exposure risk, it was of significance to scientifically assess, analyze and make a comprehensive management policy for relevant decision makers.

### 2.2. PM_2.5_ Data Source

**Figure 1:**
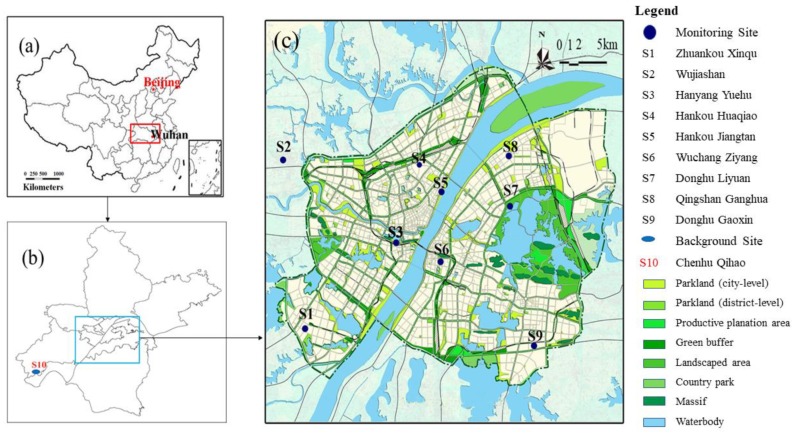
Locations of national-controlling ambient air quality monitoring sites in Wuhan: (**a**) location of Wuhan in China; (**b**) administration boundary of Wuhan and spatial distribution of nationally controlled ambient air quality monitoring sites, (S10 is Chenhu Qihao (background site)); (**c**) detailed monitoring site locations on the land-use map of Wuhan urban areas.

This study obtained data of the nationally controlled ambient air quality monitoring sites from the Wuhan Environmental Status Bulletin (2013–2017) [19]. The layout of the national air control points of Wuhan Ambient Air Quality Monitoring is shown in Figure 1. Chenhu Qihao (S10) is a background site. On the basis of data from these sites, the annual average PM_2.5_ concentration of 13 districts in Wuhan was simulated by using the built-in Voronoi neighbor averaging (VNA) interpolation method of BenMAP-CE software [24].

### 2.3. Health Impact Assessment

BenMAP-CE v1.4 was used to assess the health benefits of improving air quality in Wuhan, which is a health assessment tool for air pollution control designed by the US Environmental Protection Agency (EPA) and South China University of Technology [25,26]. The health impact function is used to assess changes in adverse health effects associated with changes in exposure to air pollution [27]. BenMAP-CE can generate the air quality map layer in the baseline scenario and control each scenario on the basis of its pollutant concentrations. Then, the air quality map layers in the above scenarios were matched with the map layer of the exposed population data. Furthermore, the health impact function is used to calculate the health benefits due to changes in pollutant concentration [28]. Since death is the most significant end point of various health effects related to PM_2.5_ pollution, this paper chooses all-cause death as the end point of health effects [29,30]. For this study, Equation (1) was used to evaluate the health benefits obtained by controlling the PM_2.5_ concentration in Wuhan [26].

(1) $${\Delta Y\;=\;Y}_{0}\left({1\;-\;e}^{{-\beta \Delta \mathrm{PM}}_{2.5}}\right)*Pop$$

where Δ*Y* (person) is avoided premature deaths; *Y*_0_ (‰) is the baseline rate of all-cause death in 2017; β is the coefficient between PM_2.5_ concentration and health effects (concentration–response coefficient); *Pop* (person) is the exposed population in 2017 (details are in Table S4); and ΔPM_2.5_ (μg/m³) is the change of annual average concentration of PM_2.5_ between different years [31]. We chose 2013 as the baseline scenario and 2017 as the control scenario to calculate the premature deaths avoided by controlling PM_2.5_ pollution in Wuhan at the district level. The data of baseline all-cause mortality rate and the exposed population of Wuhan were obtained from Wuhan statistical yearbook [22]. By comparison with other classical epidemiological studies, we derived β from meta-analysis conducted by Hoek et al. because the study was multiregional meta-analysis and had clear health-effect endpoints [32]. According to the study of Hoek et al., the relative risk (RR) of all-cause mortality was 1.0600 (95% CI: 1.0400, 1.0800) per 10 μg/m³ of long-term exposure to PM_2.5_. Epidemiological studies linking PM_2.5_ exposure to all-cause mortality are shown in Table S5, and Supplementary Materials also state details about calculating β on the basis of epidemiological study results.

### 2.4. Economic Benefits Estimation

Placing a monetary value on clean air can be essential for the evaluation of air quality improvement policies [33]. Economic benefits allow policy makers to assess the benefits of air pollution strategies from an economic perspective [34]. Monetization methods such as cost of illness (COI), the human capital approach (HCA), and willingness to pay (WTP) are often used to assess the health benefits of controlling air pollution. COI is used to calculate the cost of medical expenses caused by diseases. It is applicable to the calculation of small-scale and high-pollution areas, but it lacks accuracy for areas and cities with larger areas and more complicated pollution situations. HCA emphasizes damage caused by air pollutants to the capital embodied in workers. This method implies that the lives of the rich are more valuable to society than the lives of the poor. The value of the unemployed and the retired elderly is almost zero, and young people who die prematurely have a low value because they have not yet reached the working age and have no source of income when they die. HCA is largely contrary to our concept of sustainable development [35]. Based on the willingness to pay method, value of a statistical life (VSL) refers to the value that society is willing to pay to reduce a certain risk of death or prevent a member of society from an early death. Compared with the three methods above, VSL was selected due to its flexibility and operability [36]. The model for evaluating economic benefits by controlling PM_2.5_ is shown in Equation (2):

(2) HBE = ΔY × VSL

where Δ*Y* (person) is avoided premature deaths, *VSL* (million RMB/person) is the unit economic value of the health endpoint, and *HBE* is the sum of economic change of the health endpoint.

VSL can be obtained in the following three ways. One is to obtain first-hand data of the study area. For example, Wang and Mullahy used the contingent valuation method (CVM) to assess the willingness to pay to reduce fatal risk by improving air quality in Chongqing, China [37]. The average annual income of 500 respondents was 490 US dollars, and its VSL was 34,458 US dollars. Hammitt and Zhou conducted a survey in Beijing, Anqing and the rural areas near Anqing in 1999, with VSL values ranging from 4000 US dollars to 17,000 US dollars [38]. Secondly, relevant VSL values are obtained through meta-analysis. For example, the research results of Xu et al. showed that air pollution-related VSL in China was about 0.86 million RMB, urban VSL was about 1.59 million RMB, and rural VSL was about 0.32 million RMB [39]. The third is the result transfer method, that is, the VSL of residents in the study area in the particular year can be obtained through formula conversion [27,40]. When limited by workforce and financial resources, this method could economically and effectively obtain the VSL of the designated year in a research area [14,27]. For reflecting differences in the average income levels, it is necessary to transfer the international estimates across countries and within a country over time [27]. The initial VSL of the result transfer method could be derived from meta-analysis, the CVM, or the results transferred by other studies [17,41,42]. Because the statistical caliber of “per capita disposable income” in statistical yearbooks of different regions slightly varies, this study converted the VSL of Shanghai residents in 2017 (4.22 million RMB) on the basis of the results of Dai et al. [17]. Then, the VSL of Wuhan residents in 2017 was calculated on the basis of the Equation (3):

(3) $$VSL_{WH2017}=\;4.22\;\times \;{\left(\frac{I_{WH2017}}{I_{SH2017}}\right)}^{e}$$

where *VSL*_WH2017_ (million RMB/person) is the *VSL* of Wuhan residents in 2017; *I*_WH2017_ (RMB/person) and *I*_SH2017_ (RMB/person) are the per capita disposable income of Wuhan and Shanghai in 2017, respectively; and *e* is the elastic coefficient of WTP and is assumed to be 0.8. The number of the health impacts of PM_2.5_ concentration changes multiplied by the *VSL* of each individual were the economic benefits of controlling PM_2.5_ pollution in the study area.

## 3. Results

### 3.1. Spatio-Temporal Changes of PM_2.5_ Concentration in Wuhan

Annual average concentration changes of PM_2.5_ in Wuhan in 2013–2017 are shown in Table 1. Furthermore, on the basis of monthly average concentrations of PM_2.5_ in Wuhan, the highest and lowest values are shown in a boxplot in Figure S1. After the implementation of the Air Quality Improvement Action Plan, the annual average PM_2.5_ concentration in Wuhan significantly decreased, from 1.7 times to 0.5 times in excess of the national ambient air quality standard (35 μg/m³), and from 8.4 times to 4.3 times in excess of the air quality guidelines (AQG) set by the World Health Organization (WHO; 10 μg/m³) [43]. Although the implementation of the Plan had a good effect on reducing the PM_2.5_ exposure concentration in Wuhan, it is necessary to quantitatively assess the cost–benefit effects of its implementation and to further develop targeted improvement. Further comparison of the average annual PM_2.5_ concentration in the research area in 2013 and 2017 showed that the decrease in PM_2.5_ concentration in Wuchang District (46.7%), Jianghan District (46.0%), and Hanyang District (45.2%), Jiang’an District (44.8%), Qiaokou District (44.8%), Xinzhou District (44.8%), and Dongxihu District (44.2%) was above the city’s average (43.6%).

### 3.2. Health and Health Economic Benefits of Controlling PM_2.5_ Pollution

On the basis of Equation (1), many researchers only compared the ΔPM_2.5_ in beginning and end of their studied time lag [29,44]. The number of avoided premature deaths due to PM_2.5_ reduction in Wuhan were shown in Table 2. The total number of avoided premature deaths in these four periods were 21,384 (95% CI: 15,004 to 27,255), accounting for 7.84% of the total deaths in Wuhan between 2013 and 2017. The avoided premature deaths in Huangpi District (2842 people), Wuchang District (2722 people), Hongshan District (2670 people), and Xinzhou District (2457 people) were all over 2000 people due to the decrease of PM_2.5_ concentration. Avoided premature deaths in seven central urban districts totalled 12,657, which was 1.45 times as many as in six suburban districts. In addition to the decrease of PM_2.5_ concentration, the above results were also related to the exposed population base of the study area.

**Figure 2:**
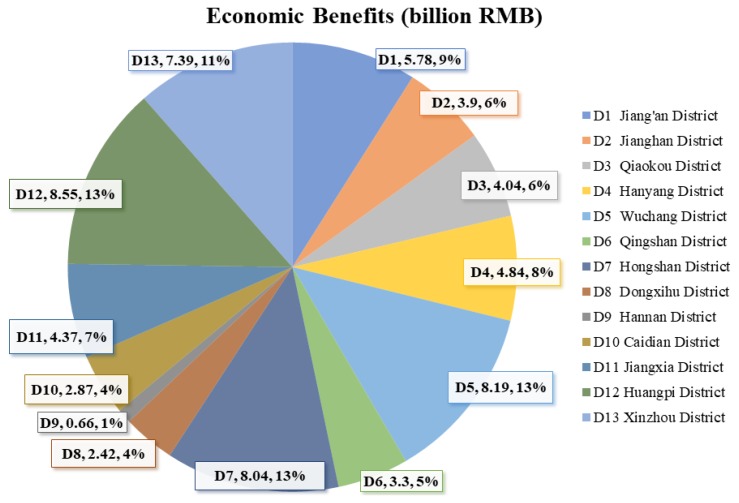
Economic benefits of PM_2.5_ concentration reduction in Wuhan between 2013 and 2017 (billion RMB). (**D1**), 5.78, 9% means that the economic benefit of Jiang’an District was 5.78 billion RMB, accounting for 9% of the total economic benefits of Wuhan.

In 2017, the value of the statistical life of residents in Wuhan was 3.01 million RMB. Combined with the premature deaths avoided by the decrease of PM_2.5_ concentration by annual estimation, the economic benefits obtained in all districts of Wuhan between 2013 to 2017 were calculated and were shown in Figure 2. The economic benefits of controlling PM_2.5_ pollution in Wuhan were 64.35 billion RMB (95% CI: 45.15 to 82.02 billion, RMB), accounting for about 4.8% (95% CI: 3.4% to 6.1%) of the city’s GDP in 2017. To the authors’ best knowledge, the Wuhan government planned to invest 28 billion RMB to work on air pollution from 2013 to 2017 [45]. According to the assessment, the economic benefits were higher than the special funds set up by the Wuhan government to improve air quality. The economic benefits gained in the central urban districts were 38.09 billion RMB, which was 11.83 billion RMB more than the benefits in suburban districts. Economic benefits in D12 (Huangpi District, 8.55 billion RMB), D5 (Wuchang District, 8.19 billion RMB), D7 (Hongshan District, 8.04 billion RMB), D13 (Xinzhou District, 7.39 billion RMB), and D1 (Jiang’an District, 5.78 billion RMB) were more than the average level in Wuhan (4.95 billion RMB). Due to it having the smallest reduction in PM_2.5_ concentration and the smallest number of exposed people, the economic benefits obtained in D9 (Hannan District, 0.66 billion RMB) were obviously lower than the average level of the whole city.

## 4. Discussion

Compared with cities that did not implement similar policies to improve air quality between 2013 and 2017, Wuhan city witnessed a larger drop in annual average concentration of PM_2.5_. For example, the annual average PM_2.5_ concentration in Kunming, Urumqi, and Wuhan city decreased by 30.95% (from 42 to 29 μg/m³), 19.54% (from 87 to 70 μg/m³), and 43.6% (from 94 to 53 μg/m³) from 2013–2017, respectively. Perhaps this comparison could serve as an indication that a region with a policy to improve air quality may see a larger drop in PM_2.5_ concentrations than a region without such a policy. During the period of implementation of the Air Improvement Action Plan, Wuhan obtained great health economic benefits due to the control of PM_2.5_. However, on the basis of the impact of climate conditions, enterprise production cycles, and other factors on air quality, the following contents of the Plan could be improved. First, the Plan only focused on the decline of PM_2.5_ annual average concentration, and did not develop a control plan concerning quarterly, monthly, and daily PM_2.5_ average concentration. This would increase the risk of residents suffering from the acute effects of PM_2.5_ in the case of a pollutant concentration surge. Second, the plan only considered establishing stricter emission standards in the city’s areas at upwind direction (Jiang’an, Qingshan, Huangpi, and Xinzhou District) when formulating emission standards. On the basis of health economics, the densely populated areas (Wuchang and Hongshan District) also need to establish stricter emission standards.

When evaluating the health benefits for controlling air pollution, one of the most important differences lies in the choice of control scenarios. Some scholars chose the ambient air quality standards (or a threshold value that does not produce health effects, or a concentration value at the clean level), others have simulated the pollutant concentrations as a control scenario on the basis of the assumed policy scenario, and others still used the actual concentrations of the evaluation years as a control scenario [10,16,46,47,48]. For example, Wu et al. took the target value of the first phase of the WHO interim target-1 (that is, China’s second-level environmental air quality standards, 35 μg/m³) as the control scenario to estimate premature deaths in China in 2013 and 2017, then subtracted the results of 2013 from the results of 2017 to obtain the number of premature deaths that could have been prevented by the implementation of the Air Pollution Prevention and Control Action Plan (2013–2017) [16]. We used the actual concentrations between 2013 and 2017 to calculate the PM_2.5_-related health benefits in Wuhan. This method could help to provide an overall estimation in the number of PM_2.5_-related avoided premature deaths in 2017 due to the Air Improvement Action Plan in Wuhan (2013–2017), and will avoid misunderstandings of the pathophysiological effects of air quality, as well as biases on short-term and long-term health impacts. In addition, Liu et al. divided 2010–2015 into five periods (2010–2011, 2011–2012, 2012–2013, 2013–2014, and 2014–2015) to evaluate the long-term health benefits of air quality improvement in China [46]. We employed a similar annual estimation method to calculate the health economic benefits since the implementation of the Air Improvement Action Plan in Wuhan—results are shown in Table S6 and Figure S2. Table S7 compares our results with other studies on the health economic benefits obtained in China during 2013 to 2017. Results vary for the study areas and estimation methods, but it is certain that the annual average concentration of PM_2.5_ in China has significantly decreased in recent years, which has led to greater health and economic benefits.

There are several uncertainties and limitations in evaluating the health economic benefits of the Air Improvement Action Plan in Wuhan City. In order to improve the evaluation method of PM_2.5_-related health economic benefits and increase public trust in this method, we also discuss the following possible solutions to the limitations [49]. In particular: (1) The district-level all-cause mortality is set to be uniform. Because variations in urbanization and PM_2.5_ pollution vary in different districts of Wuhan, in order to better account for these factors, we used district-level population data and district-level PM_2.5_ data to calculate the health benefits of each district in Wuhan. Optimally, district-level estimates should use district-level baseline all-cause mortality data, but it is difficult to obtain the all-cause mortality data for each district in Wuhan. Therefore, the present assessment assumed that the baseline all-cause mortality rates of the population in each district were the same, which led to a certain deviation in the assessment results [50]. (2) Exposed population may be undercounted. Since the baseline all-cause mortality rate of the population in Wuhan statistical yearbook was calculated on the basis of deaths in the registered population, the registered population rather than the permanent population in Wuhan was regarded as the exposed population in this study, to ensure the consistency of statistical coverage [22]. This might partly underestimate the number of avoided premature deaths in Wuhan during 2013–2017 due to the reduction of PM_2.5_ concentration. On the one hand, we found that although PM_2.5_ concentrations have declined from 2013 to 2017, the baseline all-cause mortality rates in Wuhan have not declined during the same period [22]. Other studies showed similar results [46,48,51]. Perhaps the number of people who immigrated to Wuhan and became the registered population in 2017 had an obvious impact on the city’s population structure (http://www.whzc.gov.cn/html/2017-07/61.html)—this may have an impact on all-cause mortality (Table S8). In future research, other factors that could help to decrease the PM_2.5_-related deaths, such as improvements in the education of the people, the healthcare quality, system upgrades, nutrition types, and quality of life improvements also need to be explored. (3) The various health effects of PM_2.5_ only depends on mass concentration. Since the annual average concentration of PM_2.5_ in this study was obtained from the nationally controlled ambient air quality monitoring sites, indoor and outdoor exposure were not distinguished, and the chemical compositions of indoor and outdoor air were not analyzed. Meanwhile, distinguishing between indoor and outdoor exposure of the population and studying the harm of different chemical compositions of PM_2.5_ to human health are both the focuses and difficulties of future research [52]. With continuous research refinement, the management departments could cooperate with academic research institutions to develop a localized health effects database of residents’ air pollution exposure. In the future, accurate personal evaluation can be improved by analyzing a user-level population’s exposure on the basis of an intelligent Internet of Things.

Due to the differences in PM_2.5_ pollution, exposed population, and per capita disposable income in the 13 districts of Wuhan, the health economic benefits obtained by controlling PM_2.5_ pollution in these districts were quite different. Wuchang District had the highest health economic benefits due to its large population base and significant decline in PM_2.5_ concentration. Because of a small population base and a smaller PM_2.5_ decrease than that of other districts, Hannan District had the lowest health economic benefits. The health economic benefits obtained by Wuchang District and Hannan District differed by 12-fold. For Wuhan, in terms of improving air quality, it is necessary to formulate an air quality compensation mechanism and establish hierarchical emission reduction standards and policies on the basis of air quality, population exposure, and economic development of each district.

## 5. Conclusions

After the implementation of the Air Improvement Action Plan, the areas with a high annual average drop in PM_2.5_ concentrations were concentrated in central urban areas (except Qingshan district) and Dongxihu and Xinzhou districts in the suburban areas. Due to the implementation of the Air Improvement Action Plan, the total prevented deaths in Wuhan were 21,384 (95% CI: 15,004 to 27,255) during 2013–2017, and the economic benefits add up to 64.35 billion RMB (95% CI: 45.15 to 80.02 billion RMB), accounting for 4.8% (95% CI: 3.4% to 6.1%) of the total GDP of Wuhan in 2017. Health economic benefits were more significant in densely populated areas with a large decrease in PM_2.5_ concentration. The implementation of the Plan obviously helped to improve the air quality and ensure people’s benefits in Wuhan. Furthermore, on the basis of the district-level result variation, it is recommended to establish a district-level baseline of PM_2.5_ to assist the precise health economic benefits evaluation. Meanwhile, under integrated consideration of regional differences in socioeconomic development, population size, and environment capacity, it is necessary to establish hierarchical emission reduction standards, and formulate an urban air quality compensation mechanism encouraging an optimized regional industrial/natural layout.

## Figures and Tables

**Table 1:** Annual average concentrations of PM_2.5_ in Wuhan from 2013 to 2017 (μg/m³).

District	Year					Decline in 2013–2017 (%)
	2013	2014	2015	2016	2017	
Jiang’an	97	82	69	58	53	44.8
Jianghan	95	82	71	58	51	46.0
Qiaokou	94	84	71	58	52	44.8
Hanyang	94	83	71	57	51	45.2
Wuchang	93	79	69	55	50	46.7
Qingshan	99	87	71	59	56	43.5
Hongshan	96	82	69	57	54	43.2
Dongxihu	97	86	72	58	54	44.2
Hannan	86	78	70	56	55	36.0
Caidian	88	82	70	57	55	38.0
Jiangxia	96	83	70	58	55	43.3
Huangpi	98	84	70	58	55	43.6
Xinzhou	97	82	69	58	53	44.8
Wuhan City	94	82	70	57	53	43.6

**Table 2:** Total number of PM_2.5_-related avoided premature deaths in Wuhan between 2013 and 2017 (95% confidence interval).

District	Number of PM_2.5_-Related Avoided Premature Deaths	District	Number of PM_2.5_-Related Avoided Premature Deaths
Jiang’an	1921 (1349–2445)	Dongxihu	804 (565–1024)
Jianghan	1297 (911–1650)	Hannan	219 (152–282)
Qiaokou	1342 (942–1710)	Caidian	954 (664–1224)
Hanyang	1607 (1128–2047)	Jiangxia	1451 (1016–1854)
Wuchang	2722 (1913–3645)	Huangpi	2842 (1994–3622)
Qingshan	1098 (771–1398)	Xinzhou	2457 (1725–3130)
Hongshan	2670 (1873–3405)	Wuhan City	21,384 (15,004–27,255)

## Supplementary Materials

The following are available online at https://www.mdpi.com/1660-4601/17/2/620/s1, Table S1: Ten Major tasks of Air Improvement Action Plan in Wuhan City, Table S2: Individual Air Quality index (IAQI) with corresponding pollutant concentrations, Table S3: AQI value and description, Table S4: Exposed population and baseline all-cause mortality rate in Wuhan, Table S5: Epidemiological studies linking PM_2.5_ exposure to all-cause mortality, Table S6: Total Number of PM_2.5_-related avoided premature deaths in Wuhan from 2013 to 2017, Table S7: PM_2.5_-related health economic benefits studies in China during 2013 to 2017, Table S8: Population changes in Wuhan City from 2013 to 2017; Figure S1: Highest and lowest values of monthly average concentration of PM_2.5_ in Wuhan city during the period of 2015–2017, Figure S2: Economic benefits of PM_2.5_ concentration reduction in Wuhan from 2013 to 2017 by annual estimation.
