# Supplementary material for: PM2.5-Related Health Economic Benefits Evaluation Based on Air Improvement Action Plan in Wuhan City, Middle China
Source: Int J Environ Res Public Health. 2020 Jan 18;17(2):620. doi: 10.3390/ijerph17020620 (PMC7013862; doi:10.3390/ijerph17020620)
Supplement: Supplementary file 1 [file ijerph-17-00620-s001.pdf]

$PM_{2.5}$-Related Health Economic Benefits Evaluation Based on Air Improvement Action Plan in Wuhan City, Middle China

Zhiguang Qu [1,2], Xiaoying Wang [1,2], Fei Li [1,2,3]*, Yanan Li [1,2], Xiyao Chen [1,2], Min Chen [3]**

Supporting information

Section S1. Ten Major tasks of Air Improvement Action Plan in Wuhan City

Table S1. Ten Major tasks of Air Improvement Action Plan in Wuhan City.

No.	Major tasks	Concrete measures
1	Strengthen the pollution control of enterprises and reduce the emission of air pollutants	Implement major air pollutant emission reduction projects<br>Promote the upgrading of dust removal facilities in industrial enterprises<br>Strengthen the remediation of polluting enterprises<br>Strengthen the treatment of volatile organic compounds
2	Strengthen the prevention and control of mobile source pollution and reduce vehicle pollution emissions	Improve the quality of vehicle fuel<br>Strengthen environmental protection management of motor vehicles<br>Develop green transportation<br>Promote the prevention and control of non-road emission
3	Strengthen the fine management in urban and rural areas, reduce non-point source pollution emissions	Strictly control dust pollution at the construction sites<br>Strictly control road dust pollution<br>Control dust pollution of bare ground and storage yard<br>Strengthen the prevention and control of cooking fume pollution<br>Strictly supervise the burning of straw and other wastes<br>Strictly manage dust from sand and stone mining
4	Strictly require the access system of energy conservation and environmental protection and optimize industrial development structure	Strict environmental access for construction projects<br>Speed up the elimination of backward production capacity<br>Optimize industrial development layout<br>Establish ecological industrial parks
5	Accelerate the adjustment of energy structure and improve the utilization rate of clean energy	Strictly control coal consumption<br>Speed up the construction of high pollution fuel prohibition zone<br>Improve energy utilization efficiency
6	Optimize urban spatial layout and build green ecological barrier	Optimize the layout of urban ecological space<br>Vigorously promote urban greening construction
7	Improve the emergency system of monitoring and early warning and properly deal with heavy polluted weather	Improve the monitoring and early warning system<br>Improve the emergency capacity of air pollution<br>Actively promote joint prevention and control of regional air pollution
8	Improve the system of environmental protection laws and policies, and innovate the management mechanism of environmental protection	Improve local atmospheric environmental protection laws and regulations<br>Improve environment economic policy<br>Strengthen environmental supervision and law enforcement<br>Innovate environmental management system
9	Enhance scientific and technological supports, and strengthen environmental protection industry	Improve the scientific and technological support capacity of air pollution prevention and control<br>Strengthen the research, development and promotion of air pollution control technologies in key areas<br>Vigorously cultivate energy conservation and environmental protection industry
10	Strengthen information publicity and public opinion guidance, and expand public participation	Strengthening the publicity of government information<br>Strengthen disclosure of corporate information<br>Strengthen the guidance of public opinion including carrying out various forms of publicity and education, and popularizing the knowledge of air pollution prevention and control<br>Actively expand public participation

In addition, at the government level, there are three safeguards to promote air quality improvement. Strengthen organizational leadership and improve working mechanism; establish assessment system and strengthen responsibility assessment, and increase investment in governance and provide financial guarantee [1].

Section S2 Guidelines to Calculate Air Quality index (AQI)

Table S2. Individual Air Quality index (IAQI) with corresponding pollutant concentrations.

IAQI	Pollutant Concentrations									
	SO_2 (Daily average, μg/m³)	SO_2 (hourly average, μg/m³) [a]	NO_2 (Daily average, μg/m³)	NO_2 (hourly average, μg/m³) [a]	PM_{10} (Daily average, μg/m³)	CO (Daily average, mg/m³)	CO (hourly average, mg/m³) [a]	O_3 (hourly average, μg/m³)	O_3 (8-hour moving average, μg/m³)	$PM_{2.5}$ (Daily average, μg/m³)
0	0	0	0	0	0	0	0	0	0	0
50	50	150	40	100	50	2	5	160	100	35
100	150	500	80	200	150	4	10	200	160	75
150	475	650	180	700	250	14	35	300	215	115
200	800	800	280	1200	350	24	60	400	265	150
300	1600	[b]	565	2340	420	36	90	800	800	250
400	2100	[b]	750	3090	500	48	120	1000	[c]	350
500	2620	[b]	940	3840	600	60	150	1200	[c]	500

Note
a. The hourly average concentration values of SO_2, NO_2 and CO are only used for real-time reporting, and the daily average concentration limits of corresponding pollutants need to be used in daily reports.
b. If the hourly average concentration of SO_2 is higher than 800 μg/m³, its IAQI needn't to be calculated. The IAQI of SO_2 is reported based on the daily average concentration.
c. If the 8-hour average concentration of O_3 is higher than 800 μg/m³, its IAQI needn't to be calculated. The IAQI of O_3 is reported based on the hourly average concentration.

AQI can be calculated according to Technical Regulation on Ambient Air Quality Index (HJ 633–2012) [2]. Based on the concentration limits of six pollutants in Table S1, the IAQI can be calculated by Equation (1).

$$\mathrm{IAQI_p} = \frac{\mathrm{IAQI_{Hi}} - \mathrm{IAQI_{Lo}}}{\mathrm{BP_{Hi}} - \mathrm{BP_{Lo}}}\left(\mathrm{C_p} - \mathrm{BP_{Lo}}\right) + \mathrm{IAQI_{Lo}} \quad (1)$$

where $\mathrm{IAQI_p}$ means the IAQI of pollutant p; $\mathrm{C_p}$ means the concentration of pollutant p; $\mathrm{BP_{Hi}}$ means the high value of limit value of pollutant concentration close to $\mathrm{C_p}$ in Table S1; $\mathrm{BP_{Lo}}$ means the low value of limit value of pollutant concentration close to $\mathrm{C_p}$ in Table S1; $\mathrm{IAQI_{Hi}}$ means the IAQI corresponding to $\mathrm{BP_{Hi}}$ in Table S1; $\mathrm{IAQI_{Lo}}$ means the IAQI corresponding to $\mathrm{BP_{Lo}}$ in Table S1.

Then, using Equation (2) to select the maximum value of $\mathrm{IAQI_p}$ as AQI.

$$\mathrm{AQI} = \mathrm{Max}\,\{\mathrm{IAQI_1}, \mathrm{IAQI_2}, \mathrm{IAQI_3}, \dots, \mathrm{IAQI_n}\} \quad (2)$$

where, IAQI means individual air quality index; n means different pollutants, such as $PM_{2.5}$, PM_{10}, SO_2, NO_2, O_3, CO.

Finally, the AQI level is determined according to the calculation result of Equation (2).

Table S3. AQI value and description.

AQI value	Description	AQI value	Description
0~50	Excellent	151~200	Moderate pollution
51~100	Good	201~300	Heavy pollution
101~150	Mild pollution	> 300	Serious pollution

Section S3 Exposed population and all-cause mortality rate in Wuhan

Table S4. Exposed population and all-cause mortality rate in Wuhan.

	Exposed population (person)				
	2013	2014	2015	2016	2017
Jiang’an	700,179	711,084	719,531	726,209	741,784
Jianghan	485,618	486,676	486,430	486,214	496,289
Qiaokou	528,649	527,593	526,494	524,620	528,604
Hanyang	567,230	576,536	585,373	595,834	631,185
Wuchang	1,086,411	1,076,733	1,056,137	1,041,746	1,044,072
Qingshan	442,347	438,525	433,676	428,816	426,289
Hongshan	927,275	936,139	948,785	964,851	1,073,545
Dongxihu	276,445	283,103	288,541	296,479	312,834
Hannan	111,562	112,889	113,189	113,891	114,288
Caidian	449,084	454,810	456,551	459,923	461,772
Jiangxia	583,623	588,943	590,510	598,280	611,436
Huangpi	1,112,543	1,121,602	1,124,832	1,132,828	1,133,207
Xinzhou	949,527	958,484	962,617	968,759	961,212
Wuhan City	8,220,493	8,273,117	8,292,666	8,338,450	8,536,517
All-cause mortality rate (‰)	4.98	4.97	5.75	5.44	11.62

Section S4 Relative Risk (RR) associated $PM_{2.5}$ exposure to all-cause mortality

Table S5. Epidemiological studies linking $PM_{2.5}$ exposure to all-cause mortality.

RR (95% CI) (10 μg/m³)	Study area	Reference
1.0036 (1.0010, 1.0061)	Shanghai, China	Kan et al., 2007 [3]
1.0020 (1.0007, 1.0033)	Xi’an, China	Huang et al., 2012 [4]
1.0088 (1.0030, 1.0416)	5 urban city districts and 2 rural counties in Beijing, Tianjin, and Hebei Province, China	Zhou et al., 2015 [5]
1.0090 (0.9970, 1.0180)	31 cities of 16 provinces, China	Cao et al., 2011 [6]
1.1300 (1.0400, 1.2300)	Six US cities	Dockery et al., 1993 [7]
1.1600 (1.0700, 1.2600)	Six US cities	Laden et al., 2006 [8]
1.1400 (1.0700, 1.2200)	Six US cities	Lepeule et al., 2012 [9]
1.0600 (1.0400, 1.0800)	North America (America and Canada), Asia (China), Europe (Netherlands and Italy), Oceania (New Zealand)	Hoek et al., 2013 [10]

By searching the studies on the health effects of atmospheric $PM_{2.5}$ at home and abroad, Table S4 summarizes the results of relatively representative studies on the relative risk (RR) of all-cause death related to $PM_{2.5}$. In China, there are few studies investigating the concentration-response relationship between $PM_{2.5}$ exposure and all-cause death, some of which were conducted for specific regions [3-5]. Since $PM_{2.5}$ was not monitored in China during the study's cohort period, Cao et al. converted PM_{10} concentrations to $PM_{2.5}$ concentrations (conversion as $PM_{2.5}/PM_{10}\approx0.65$), and then analyzed the concentration-response between $PM_{2.5}$ exposure and health effect endpoints [6]. Hoek et al. conducted a multi-regional meta-analysis and the study had clear health effect endpoints, they reported that excess risk (ER) per 10 μg/m³ increase in $PM_{2.5}$ exposure was 6% (95% CI: 4,8%) increased for all-cause mortality [10]. That suggested a Relative Risk (RR) of 1.0600 with respect to all-cause mortality of long-term exposure to $PM_{2.5}$ [11]. Therefore, this study selects Hoek's findings and calculates the concentration-response

coefficients (β) associated with $PM_{2.5}$ exposure based on equation (3) [12].

$$\beta = \frac{\ln(RR)}{\triangle PM} \quad (3)$$

where $\Delta PM_{2.5}$ is the air quality change. For example, if the epidemiological study reported result like "The excess risk (ER) per 10 μg/m³ increase in $PM_{2.5}$ exposure was 6% (95% CI: 4,8%) increased for all-cause mortality", that means △PM is 10 and the value of RR is 1.0600 (95% CI: 1.0400, 1.0800), so β is 0.005827.

Section S5 Highest and lowest values of $PM_{2.5}$ concentration in Wuhan

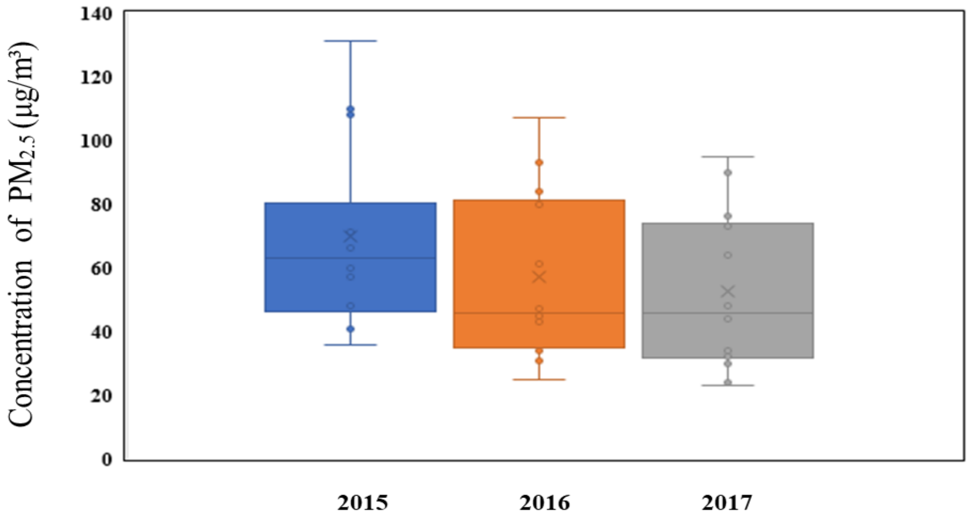

Figure S1. Highest and lowest values of monthly average concentration of $PM_{2.5}$ in Wuhan city during the period of 2015–2017.

Section S6 Impact of population structure on evaluating the long-term health economic benefits of controlling air pollution

Table S6. Total number of $PM_{2.5}$-related avoided premature deaths in Wuhan from 2013 to 2017 by annual estimation method (95% confidence interval).

District	Time Periods				
	2013–2014	2014–2015	2015–2016	2016–2017	Total
Jiang'an	275 (188-359)	268 (183-350)	266 (182-349)	96 (65-126)	905 (618-1184)
Jianghan	174 (119-228)	160 (109-209)	198 (135-259)	99 (67-130)	631 (431-826)
Qiaokou	149 (102-196)	190 (130-249)	225 (154-294)	100 (68-132)	664 (454-870)
Hanyang	175 (120-230)	189 (129-247)	261 (179-341)	110 (74-144)	735 (502-962)
Wuchang	429 (294-561)	316 (216-414)	458 (314-599)	179 (121-235)	1382 (944-1809)
Qingshan	150 (102-196)	188 (129-245)	177 (121-232)	37 (25-49)	552 (377-722)
Hongshan	354 (242-463)	344 (235-449)	364 (248-476)	80 (54-106)	1142 (780-1494)
Dongxihu	89 (61-117)	111 (76-145)	121 (83-159)	40 (27-53)	361 (247-473)
Hannan	26 (17-34)	26 (18-34)	50 (34-65)	4 (3-5)	106 (71-138)

Caidian	80 (54-105)	156 (107-204)	192 (131-251)	30 (20-39)	458 (312-599)
Jiangxia	202 (138-264)	189 (129-247)	240 (164-314)	54 (36-71)	685 (467-896)
Huangpi	408 (279-534)	400 (273-523)	450 (307-588)	122 (83-161)	1380 (942-1806)
Xinzhou	366 (250-478)	377 (258-492)	371 (253-485)	85 (57-112)	1199 (818-1567)
Wuhan City	2877 (1966-3765)	2912 (1990-3808)	3373 (2305-4412)	1035 (702-1363)	10,200 (6963-13,348)

From 2013 to 2017, the avoided premature deaths in Wuchang District (1382 people), Huangpi District (1380 people), Xinzhou District (1199 people) and Hongshan District (1142 people) were all over 1000 people due to the decrease of $PM_{2.5}$ concentration. The number of avoided premature deaths in seven central urban districts was 6011 which was 1.43 times as those in the suburban districts. The total number of $PM_{2.5}$-related avoided premature deaths in Wuhan from 2013 to 2017 was added up to 10,200 (95% CI: 6963 to 13,348), these results were smaller than the estimation that setting 2013 as baseline scenario and 2017 as control scenario in this study (21,384, 95% CI: 15,004 to 27,255). Through continuous exploration, we found that the most likely cause of the problem lies in the great change of all-cause mortality rate in Wuhan in 2017, and the change was not only affected by the improvement of air quality, but also greatly influenced by the net migrating rate in Wuhan (Table S6). If the net migrating rate > 0, means the number of immigrating populations > the number of emigrating populations in a specific period of the area. Thanks to the city's talent introduction policies, compared with 2013–2016, the number of people who immigrated to Wuhan and became the registered population in 2017 had an obvious impact on the city's population structure (http://www.whzc.gov.cn/html/2017-07/61.html). The possibility is not ruled out that people who obtained household registration in Wuhan in 2017 also lived in the city from 2013 to 2016. Therefore, setting 2013 as baseline scenario and 2017 as control scenario could contribute to reduce the uncertainty caused by population migration in a particular period.

Combined with the premature deaths avoided by the decrease of $PM_{2.5}$ concentration from 2013 to 2017, the economic benefits obtained in all districts of Wuhan were calculated and were shown in Figure S2. The economic benefits of controlling $PM_{2.5}$ pollution in Wuhan was 30.70 billion RMB (95% CI: 20.95 to 40.17 billion), accounting for about 2.3% (95% CI: 1.6% to 3.1%) of the city's GDP in 2017. The economic benefits gained in the central urban districts were 18.31 billion RMB, which was 5.92 billion RMB more than the benefits in suburban districts. Economic benefits in D5 (Wuchang District, 4.16 billion RMB), D12 (Huangpi District, 4.15 billion RMB), D13 (Xinzhou District, 3.61 billion RMB), D7 (Hongshan District, 3.44 billion RMB) and D1 (Jiang'an District, 2.72 billion RMB) were more than the average level in Wuhan (2.36 billion RMB).

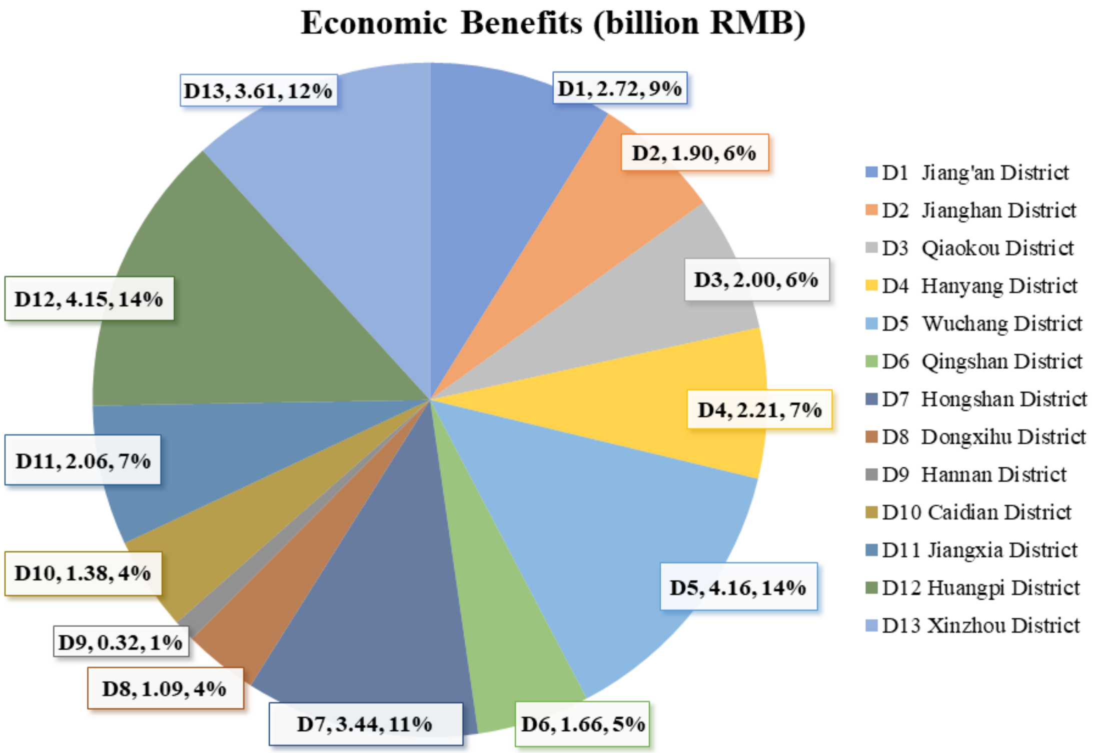

Figure S2. Economic benefits of $PM_{2.5}$ concentration reduction in Wuhan from 2013 to 2017 by annual estimation (billion RMB). D1, 2.72, 9% means that the economic benefit of Jiang'an District was 2.72 billion RMB, accounting for 9% of the total economic benefit of Wuhan.

Section S7 Comparison with other similar researches

Table S7. $PM_{2.5}$-related health economic benefits studies in China during 2013 to 2017.

Study Area	Evaluation period	$PM_{2.5}$ decline	Number of avoidable premature death	Economic benefits	Reference
China	2013–2017	39.5%	214,821	210.14 billion US$	[13]
China	2013–2017	Decline varied from province to province	60,213	54.97 billion RMB	[14]
China	2013–2017	Decline varied from province to province	718,000	3762 billion RMB	[15]
Pearl River Delta	2013–2015	28%	3800	1300 billion US$	[16]
Shanghai	2013–2017	38.3% (population-weighted annual average concentrations of $PM_{2.5}$)	3439	11.841 billion RMB	[17]
This Study	2013–2017	43.6%	21,384	64.35 billion RMB	

Table S8. Population changes in Wuhan City from 2013 to 2017.

Item	2013	2014	2015	2016	2017
Exposed Population (person)	8,220,493	8,273,117	8,292,666	8,338,450	8,536,517
Number of Births (person)	92,701	100,784	105,169	95,436	131,409
Birth Rate (‰)	11.28	12.22	12.70	11.48	15.57
Number of Deaths (person)	40,941	41,007	47,637	45,259	98,029
All-cause Mortality Rate (‰)	4.98	4.97	5.75	5.44	11.62
Net Migrating Population (person)	-25,132	360	-14,762	-2430	166,922
Net Migrating Rate (‰)	-3.06	0.04	-1.78	-0.29	19.55

References

1. the People's Government of Wuhan Municipality. Air Improvement Action Plan in Wuhan City (2013–2017). Available online: http://www.wh.gov.cn/whszfwz/xwxx/zfgb/201406/P020150313423387265894.pdf. (accessed on 30 June 2018). (in Chinese)
2. Ministry of Ecology and Environment of the People's Republic of China. HJ 633-2012 Technical Regulation on Ambient Air Quality Index (on trail); China Environmental Science Press: Beijing, China, 2012. (In Chinese)
3. Kan, H.D.; London, S.J.; Chen, G.H.; Zhang, Y.H.; Song, G.X.; Jiang, L.L.; Zhao, N.Q.; Chen, B.H. Differentiating the effects of fine and coarse particles on daily mortality in Shanghai, China. *Environ. Int.* **2007**, *3*, 376–384.
4. Huang, W.; Cao, J.; Tao, Y.; Dai, L.; Lu, S.; Hou, B. Seasonal variation of chemical species associated with short-term mortality effects of $PM_{2.5}$ in Xi'an, a central city in China. *Am. J. Epidemiol.* **2012**, *175*, 556–566.
5. Zhou, M.; He, G.; Fan, M.; Wang, Z.; Liu, Y.; Ma, J.; Ma, Z.; Liu, J.; Liu, Y.; Wang, L.; Liu, Y. Smog episodes, fine particulate pollution and mortality in China. *Environ. Res.* **2015**, *136*, 396–404.
6. Cao, J.; Yang, C.; Li, J.; Chen, R.; Chen, B.; Gu, D.; Kan, H. Association between long-term exposure to outdoor air pollution and mortality in China: A cohort study. *J. Hazard. Mater.* **2011**, *186*, 1594–1600.
7. Dockery, D.W.; Pope, C.A.; Xu, X.; Spengler, J.D.; Ware, J.H.; Fay, M.E.; Ferris, B.G., Jr.; Speizer, F.E. An association between air pollution and mortality in six U.S. cities. *N. Engl. J. Med.* **1993**, *329*, 1753–1759.
8. Laden, F.; Schwartz, J.; Speizer, F. E.; Dockery, D. W. Reduction in fine particulate air pollution and mortality: Extended follow-up of the Harvard Six Cities study. *Am. J. Respir. Crit. Care Med.* **2006**, *173*, 667-72.
9. Lepeule, J.; Laden, F.; Dockery, D.; Schwartz, J. Chronic exposure to fine particles and mortality: An extended follow-up of the Harvard Six Cities study from 1974 to 2009. *Environ. Health Perspect.* **2012**, *120*, 965–970.
10. Hoek, G.; Krishnan, R.M.; Beelen, R.; Peters, A.; Ostro, B.; Brunekreef, B.; Kaufman, J.D. Long-term air pollution exposure and cardio-respiratory mortality: A review. *Environ. Health* **2013**, *12*, 43.
11. Belis, C.; Pisoni, E.; Degraeuwe, B.; Peduzzi, E.; Thunis, P.; Monforti, F.; Guizzardi, D., Urban pollution in the Danube and Western Balkans regions: The impact of major $PM_{2.5}$ sources. *Environ. Int.* **2019**, *133*, 105158.
12. US EPA. BenMAP-CE User's manual. Available online: https://www.epa.gov/sites/production/files/2015-04/documents/benmap-ce_user_manual_march_2015.pdf (accessed on 10 July 2018)
13. Zou, B.; You, J.; Lin, Y.; Duan, X.; Zhao, X.; Fang, X.; Campen, M. J.; Li, S., Air pollution intervention and life-saving effect in China. *Environ. Int.* **2019**, *125*, 529-541.
14. Wu, W.; Xue, W.; Wang, Y.; Lei, Y.; Feng, T.; Cai, Z. Health Benefit Evaluation for Air Pollution Prevention and Control Action Plan in China. *Environmental Science.* **2019**, *07*, 1-9. (in Chinese)
15. Ding, D.; Xing, J.; Wang, S.; Liu, K.; Hao, J., Estimated Contributions of Emissions Controls, Meteorological Factors, Population Growth, and Changes in Baseline Mortality to Reductions in Ambient $PM_{2.5}$ and $PM_{2.5}$-Related Mortality in China, 2013–2017. *Environ. Health Persp.* **2019,** *127*, 067009.

16. Li, J.; Zhu, Y.; Kelly, J. T.; Jang, C. J.; Wang, S.; Hanna, A.; Xing, J.; Lin, C. J.; Long, S.; Yu, L., Health benefit assessment of $PM_{2.5}$ reduction in Pearl River Delta region of China using a model-monitor data fusion approach. *J. Environ. Manage.* **2019**, *233*, 489-498.
17. Dai, H.; An, J.; Li, L.; Huang, C.; Yan, R.; Zhu, S.; Ma, Y.; Song, W.; Kan, H.. Health Benefit Analyses of the Clean Air Action Plan Implementation in Shanghai. *Environ. Sci.* **2019**,40, 24-32. (in Chinese)
